# Isolation, genomic characterization, and mushroom growth-promoting effect of the first fungus-derived *Rhizobium*

**DOI:** 10.3389/fmicb.2022.947687

**Published:** 2022-07-22

**Authors:** Zhongyi Hua, Tianrui Liu, Pengjie Han, Junhui Zhou, Yuyang Zhao, Luqi Huang, Yuan Yuan

**Affiliations:** ^1^National Resource Center for Chinese Materia Medica, China Academy of Chinese Medical Sciences, Beijing, China; ^2^School of Pharmaceutical Sciences, Peking University, Beijing, China

**Keywords:** bacterial genome, *Rhizobium*, *Polyporus umbellatus*, mushroom growth-promoting bacteria, sclerotia

## Abstract

*Polyporus umbellatus* is a well-known edible and medicinal mushroom, and some bacteria isolated from mushroom sclerotia may have beneficial effects on their host. These mushroom growth-promoting bacteria (MGPBs) are of great significance in the mushroom production. In this work, we aimed to isolate and identify MGPBs from *P. umbellatus* sclerotia. Using the agar plate dilution method, strain CACMS001 was isolated from *P. umbellatus* sclerotia. The genome of CACMS001 was sequenced using PacBio platform, and the phylogenomic analysis indicated that CACMS001 could not be assigned to known *Rhizobium* species. In co-culture experiments, CACMS001 increased the mycelial growth of *P. umbellatus* and *Armillaria gallica* and increased xylanase activity in *A. gallica*. Comparative genomic analysis showed that CACMS001 lost almost all nitrogen fixation genes but specially acquired one redox cofactor cluster with *pqqE*, *pqqD*, *pqqC*, and *pqqB* involved in the synthesis of pyrroloquinoline quinone, a peptide-derived redox participating in phosphate solubilization activity. Strain CACMS001 has the capacity to solubilize phosphate using Pikovskaya medium, and *phnA* and *phoU* involved in this process in CACMS001 were revealed by quantitative real-time PCR. CACMS001 is a new potential *Rhizobium* species and is the first identified MGPB belonging to *Rhizobium.* This novel bacterium would play a vital part in *P. umbellatus*, *A. gallica*, and other mushroom cultivation.

## Introduction

Cultivated mushrooms are well-known food and medicinal resources throughout the world (Roncero-Ramos and Delgado-Andrade, 2017). The total value of mushroom products has exceeded 24 billion USD and provided living for more than 25 million mushroom farmers in 2011 (Zhang et al., 2014). A variety of bacterial and fungal endophytes have been identified to play critical roles in different phases of the mushroom life cycle, covering spore germination, hyphal elongation and proliferation, and fruiting body formation (Kertesz and Thai, 2018). Therefore, it is of great significance to investigate beneficial microorganisms that could improve the productivity of mushrooms. Among them, mushroom growth-promoting bacteria (MGPBs) are gaining more interests (Carrasco and Preston, 2020).

Many MGPB strains have been isolated, and their beneficial effect involves two stages: fungal mycelial growth and fructification. For mycelium, a batch of MPGBs could promote mycelial growth. It has been reported that *Pseudomonas putida* Bt4, *Glutamicibacter arilaitensis* MRC119, *Paenibacillus pectinilyticus*, and *Paenibacillus taichungensis* could promote the growth of *Agaricus bisporus* (Zarenejad et al., 2012), *Pleurotus florida* (Kumari and Naraian, 2021), *Pleurotus ostreatus* (Suarez et al., 2020), and *Tricholoma matsutake* (Oh and Lim, 2018), respectively. Among them, a field experiment using *P. putida* Bt4 increased *A. bisporus* yield by 14.04%, indicating the huge potential of applying these MGPBs in the mushroom industry. For fructification, it has been shown that *Micromonospora lupini* could reduce *A. bitorquis* spawn running time (Suarez et al., 2020). Another example is that *P. putida* could stimulate primordia to induce *A. bitorquis* fructification, which is of great importance for the *A. bisporus* industry as *A. bisporus* cannot produce primordia in sterile casing layers or petri dishes (Colauto et al., 2016). Currently known mechanisms for MGPBs promoting fungal growth include (1) conducting as an important source of nitrogen by releasing ammonium and urea (McGee, 2018); (2) shortening the time of compost preparation by providing enzymes responsible for cellulose, hemicellulose, and lignin degradation (Vieira et al., 2019); (3) producing phytohormones, especially indole-3-acetic acid (IAA; Suarez et al., 2020); (4) degrading volatile fungal growth inhibitor (Zarenejad et al., 2012); and (5) acting as biocontrol agents (Pandin et al., 2018).

*Polyporus umbellatus* is a well-known edible and medicinal mushroom that contains bioactive compounds with immunomodulatory activity, antioxidant activity, and anti-cancer activity (He et al., 2016; Liu et al., 2020). In our previous genome survey of *P. umbellatus* (unpublished data), we noticed some abnormal reads belonging to rhizobia (Supplementary Figure 1), suggesting that some rhizobia may colonize inside the *P. umbellatus* sclerotia. The production of the *P. umbellatus* sclerotia depends on the symbiotic relationship with *Armillaria* species (Liu et al., 2017), who are also known as editable mushrooms. To our knowledge, only one MGPB belonging to rhizobia has been reported (Zhu et al., 2013). Despite the fact that several bacteria have been isolated from other fungi sclerotia (Luo et al., 2021; Nonoyama and Narisawa, 2021), none of them have been proved to be MGPB.

In this study, a strain was isolated from *P. umbellatus* sclerotia and identified as a putative new species belonging to *Rhizobium.* The co-culture experiment demonstrated the isolated strain could promote the growth of *P. umbellatus* and *Armillaria gallica.* We further shed light on the potential mechanisms underlying the interaction between the isolated strain and *P. umbellatus* and *A. gallica*.

## Materials and methods

### Isolation and culture conditions

*Polyporus umbellatus* (Pers.) Fries sclerotia were collected from a broad-leaved mixed forest (33°309″N, 108°828″E) in Ningshan County, Shaanxi Province, China, in October 2018. No specific permission was required for collecting this material. The sclerotia were washed with water and soaked in 75% ethanol three to 5 min. The sclerotia were then flushed by sterile water, soaked in 2% sodium hypochlorite for 7 min, and flushed again with sterile water five times. Sterile water from the last rinse was coated on the yeast morphology agar (YMA) medium, composed of 0.2 g⋅L^–1^ K_2_HPO_4_, 0.2 g⋅L^–1^ MgSO_4_, 0.2 g⋅L^–1^ mannitol, 0.3 g⋅L^–1^ yeast extract, 0.05 g⋅L^–1^ NaCl, 10 g⋅L^–1^ agar, and cultured at 28^°^C for 2 days to confirm the surface of sclerotia was sterilized completely.

The sterilized sclerotia were ground, and the suspension was coated on YMA medium and then inversion cultured at 28^°^C for 2–5 days. After several times of culturing and isolating, one strain was isolated.

### Evaluation of *Rhizobium* species effects on the growth of *P. umbellatus* and *A. gallica*

The effects of three *Rhizobium* species on the growth of *P. umbellatus* and *A. gallica* were evaluated. In addition to the isolated strain, *Rhizobium grahamii* CCGE502^T^ and *Rhizobium leguminosarum* bv. *viciae* USDA 2370^T^ were purchased from China General Microbiological Culture Collection Center. The bacteria were grown overnight to OD_600_ = 0.7 on LB medium at 28^°^C 200 rpm. *P. umbellatus* and *A. gallica* were grown on modified plates (20 g⋅L^–1^ glucose, 5 g⋅L^–1^ yeast extract fermentation, 1 g⋅L^–1^ KH_2_PO_4_, 1 g⋅L^–1^ MgSO_4_, and 15 g⋅L^–1^ agar). The experiment was conducted in three identical replicates (*n* = 3).

For *P. umbellatus*, co-culture plates were prepared by transferring 6-mm*-*diameter *P. umbellatus* mycelial disks from the actively growing colonies to the center of the new plates. The bacteria were then streaked at the corner of the plates, 2 cm away from the fungal disk. The plates without the bacterial isolates were used as control. All plates were incubated at 25^°^C under dark conditions for 20 days. The mycelial growth was measured by the diameter of the colony. To eliminate the irregular colony shape influence on measurement, two perpendicular diameters were measured for each plate, and the average of two measurements was used (Hendricks et al., 2017). Differences in growth between treated and control plates (I) were calculated by percentage changed using the following formula:


$$I=\frac{R_{b}-R_{0}}{R_{c}-R_{0}}\times 100\%$$

where *R*_0_ is the initial inoculation diameter (i.e., 6 mm), *R*_*c*_ is the radial growth of fungi in control plates, and *R*_*b*_ is the radial growth of fungi in test plates.

For *A. gallica*, a rhizomorph of 2 mm length from the actively growing colonies was transferred to the center of new petri dishes. A measure of 100 μL bacterial solution was then streaked at the corner of the petri dishes, 2 cm away from the fungal rhizomorph. Plates without the bacterial isolates were used as control. All plates were incubated at 25^°^C under dark conditions for 10 days. The fresh weight and the activity of three extracellular enzymes (i.e., xylanase, laccase, and cellulase) of *A. gallica* mycelium were measured. Briefly, the solid medium surrounding the mycelium was collected and centrifuged (14,000 rpm, 15 min, and 4^°^C). Then 1 mL of the supernatant was then diluted to a total volume of 10 mL and used as an enzyme solution to measure the enzyme activity. The activities of cellulase and xylanase were determined using a previously reported method (Miller, 1959). To measure cellulase activity, 100 μL of enzyme solution, 1.5 mL of 1% CMC-Na solution (in 0.1 M sodium acetate buffer, pH 4.6), and 1.5 mL of 3, 5-dinitrosalycilic acid (DNS) solution (40 mM DNS, 400 mM NaOH, 1 M K-Na tartrate) were incubated at 100^°^C for 5 min and then diluted with ultrapure water to a total volume of 10 mL. The absorbance at 500 nm (A500) was measured, and the reducing ability was evaluated with a standard curve obtained with glucose (0.01–0.09 g⋅L^–1^). The xylanase activity was determined using the same procedure, using 1.5 mL of 1% xylose solution, and the standard curve was obtained using xylose (0.01–0.09 g⋅L^–1^). The reaction mixture used to measure laccase activity contained 100 mM citric acid phosphate disodium hydrogen phosphate buffer—pH 5.0, 0.2 mM 2, 2’-azino-bis (3-ethylbenzthiazoline-6-sulfonic acid; ABTS), and 0.5 mL of enzyme solution for a total volume of 2 mL. The laccase activity was measured at 420 nm over time (Sterkenburg et al., 2018).

### Identification of CACMS001 using morphological, biochemical, and fatty acid profile

Cell morphology was examined using a light microscope (CX21; Olympus). Gram staining was carried out using the Gram Stain Kit (Solarbio, Beijing, China). The basic biochemical characteristics were investigated on API-50CH test strips (bioMérieux, Marcy-I’Etoile, France).

For cellular fatty acid analysis, *Rhizobium* sp. CACMS001 was grown on YMA for 2 days at 28^°^C. The cultures were harvested, and fatty acid methyl esters were prepared and separated using methods described by Sasser (1990) and were identified with the MIDI Sherlock Microbial Identification System.

### Genome sequencing, assembly, and genome annotation

Genomic DNA was extracted using the sodium dodecyl sulfate method (Goldenberger et al., 1995). The library for single-molecule real-time (SMRT) sequencing was constructed with an insert size of 10 kb using the SMRTbell TM Template Kit, version 1.0. The genomic DNA of *Rhizobium* sp. CACMS001 was sequenced using the PacBio Sequel platform at the Beijing Novogene Bioinformatics Technology Co., Ltd. The genome was further assembled using the software SMRT Link v5.0.1 (Pacific Biosciences, CA, United States).

Open reading frames were predicted using Prokka 1.14 (Seemann, 2014). For non-coding genomic features, transfer RNA (tRNA), ribosomal RNA (rRNA), and small RNA (sRNA) genes were identified by aragorn version 1.2 (Laslett and Canback, 2004), barrnap version 0.9, and Rfam (Kalvari et al., 2021), respectively. The protein-coding gene functions were annotated by eggNOG webserver (Huerta-Cepas et al., 2019) using the Clusters of Orthologous Groups (COG) database (Galperin et al., 2015), Gene Ontology (GO) database^1^, and the Kyoto Encyclopedia of Genes and Genomes (KEGG) database^2^. For COG ID with more than one functional category, each functional category was assigned with equal partial weights (Kim et al., 2017).

Nitrogen-fixing and nodulation genes in *Rhizobium* species were annotated by performing bidirectional BLAST with an *E*-value cut-off at 1e-5 against a database of curated *fix*, *nif*, and *nod* protein sequences retrieved from GenBank and Uniprot (Supplementary Table 1). Lignin-modifying enzymes (LMEs), cellulases, and hemicellulases were identified based on Carbohydrate-Active Enzymes (CAZymes; Drula et al., 2022) using dbCAN2 webserver (Zhang et al., 2018) according to the method previously described (Bredon, 2018).

The secondary metabolite biosynthetic gene clusters of the *Rhizobium* species were predicted using antiSMASH 5.0 webserver (Blin et al., 2019). In addition, the IAA biosynthetic cluster was identified according to nucleotide sequences reported previously (Leveau and Gerards, 2008). 1-Aminocyclopropane-1-carboxylic acid (ACC) deaminase (AcdS) coding gene was identified using BLAST according to AcdS protein sequence reported in *H. saturnus*, with an *E*-value cut-off at 1e-5 (Yao et al., 2000). To identify antimicrobial peptides (AMPs) in *Rhizobium* species, AMP sequences were downloaded from the LAMP2 database (Ye et al., 2020) and then tidied to create a local database for performing BLAST analysis, with an *E*-value cut-off at 1e-5. Gene coding proteins with a length greater than 100 were excluded from the AMP results.

The whole genome sequence data reported in this article have been deposited in the Genome Warehouse in National Genomics Data Center (Chen et al., 2021; CNCB-NGDC Members and Partners, 2022), Beijing Institute of Genomics, Chinese Academy of Sciences/China National Center for Bioinformation, under accession number GWHABKA00000000 that is publicly accessible at https://ngdc.cncb.ac.cn/gwh.

### Phylogenetic analysis of the strain CACMS001

The 16S rRNA gene of strain CACMS001 was amplified using the universal primers 27F and 1492R according to Stackebrandt and Goodfellow (1991) and then compared with sequences of type strains using EzBioCloud (Yoon et al., 2017). The top 20 sequences with the highest similarity were retrieved for constructing phylogenetic trees. Multilocus sequence analysis (MLSA) was performed using four housekeeping genes, that is, *recA*, *atpD*, *glnII*, and *rpoB* previously used in taxonomic studies of *Rhizobium* (Lopez-Lopez et al., 2012). These gene sequences were retrieved from the whole-genome sequence, and node support was evaluated with bootstrap analysis using 1,000 ultrafast bootstraps (Hoang et al., 2018). *Bradyrhizobium diazoefficiens* USDA 110 were used as outgroups. All sequence alignments were conducted using Muscle 5 (Edgar, 2021) and trimmed using trimAl (Capella-Gutiérrez et al., 2009). The models for phylogenetic trees were determined by ModelFinder (Kalyaanamoorthy et al., 2017). The phylogenetic trees were constructed by RAxML-NG (Kozlov et al., 2019). The similarities between sequences were calculated using MEGA X (Kumar et al., 2018). Detailed nucleotide sequences and genomic information used in the phylogenetic analysis are listed in Supplementary Tables 2, 3. Non-type strains of some species were used because the genomes of their type strains remained unsequenced. The average nucleotide identities (ANIs) were calculated by JspeciesWS (Richter et al., 2016), including ANI based on BLAST (ANIb), and ANI based on MUMmer (ANIm). The digital DNA-DNA hybridization (dDDH) was calculated by GGDC 3.0 (Meier-Kolthoff et al., 2022).

### Comparative genomic analysis of *Rhizobium* species

*Rhizobium* sp. CACMS001, three *R. grahamii* strains, and *Rhizobium metallidurans* DSM 26575 were selected for comparative genomic analysis. The homologous genes (including orthologs and paralogs) in all genomes were first classified using OrthoFinder2 (Emms and Kelly, 2019). The positive selection analysis was performed using 2,539 single-copy families. Each single-copy ortholog sequences were aligned using Muscle. The Ka/Ks ratio was calculated using the PAML package (Yang, 2007).

### Evaluation of *Rhizobium* species phosphate solubilization activity

The screening for phosphate solubilization was performed using the standard method (Nautiyal, 1999). Briefly, 10 μL of bacterial suspension was inoculated on Pikovskaya (PVK) medium plates. The plates were incubated at 25^°^C for 5 days, and the formation of halo zones around the colonies was observed. The experiment was conducted in three replicates (*n* = 3) and used uninoculated media as control.

### Quantitative real-time PCR

The copy number of genes related to the phosphate metabolic process in strain CACMS001 were detected by quantitative real-time PCR (qPCR). The strain CACMS001 was precultured in yeast mannitol broth (YMB) medium, containing all ingredients of YMA except agar, at 28^°^C and 200 rpm for 48 h. A volume of 10 μL of the bacterial suspension was inoculated into a 50-mL conical flask containing 10 mL of distinct broth media differing with regard to the P source: (1) YMB (control); (2) YMB with 10 g⋅L^–1^ of Ca_3_ (PO_4_)_2_ (IPi); (2) YMB with 5 mmol⋅L^–1^ of K_2_HPO_4_ (LPi); and (3) YMB with 10 mmol⋅L^–1^ of K_2_HPO_4_ (HPi).

The total RNA of strain CACMS001 was extracted using TRIzol (Invitrogen, CA, United States), and cDNA was synthesized using the TransScript II First-Strand cDNA Synthesis Super Mix Kit (TransGen Biotech, Beijing, China) according to the manufacturer’s instructions. A Tip Green qPCR SuperMix Kit (TransGen Biotech, Beijing, China) was used to conduct qPCR on a LightCycler480 II system (Roche, Penzberg, Germany).

The genes related to the phosphate metabolic process from different pathways were selected for this assay, including *gcd* from the inorganic P solubilization pathway; *phnA*, *phnH*, and *phoA* from the organic P mineralization pathway; *phoR*, *phoU* from the P regulation pathway; and *pstB* and *ugpA* from P transportation pathway. The primers of qPCR analysis are listed in Supplementary Table 4. Delta Ct values were calculated by subtracting Ct values for 16S rRNA gene and relative expression derived using the delta-delta Ct method.

## Results

### Genome features

**FIGURE 1:**
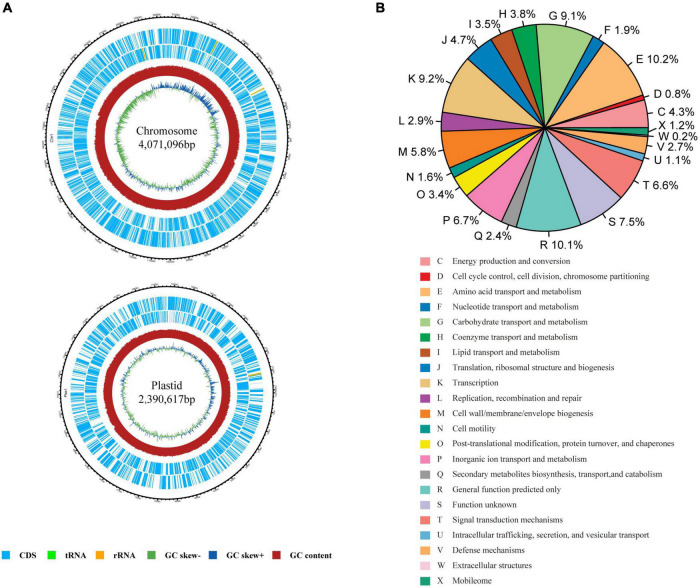
Genome characteristics of *Rhizobium* sp. CACMS001. **(A)** Graphical genetic features of *Rhizobium* sp. CACMS001. From outside to the center: genes on the forward strand, genes on the reverse strand, G+C content, and GC skew. **(B)** The relative proportion of *Rhizobium* sp. CACMS001 genes in 22 COG categories.

A total of 148,699 reads with an average length of 7,723 bp were obtained using PacBio Sequel II. The genome of the strain was found to contain one chromosome of 4,071,096 bp and one plasmid of 2,390,617 bp (Figure 1A). In total, 6,171 coding sequences (CDS) were predicted with 3,939 CDS in the chromosome and 2,232 CDS in the plasmid. Among them, 5,069 CDS were assigned into the functional categories of COG database (Figure 1B). The top five categories are E (amino acid transport and metabolism; 10.20%), R (general function prediction; 10.14%), K (transcription; 9.20%), G (carbohydrate transport and metabolism; 9.08%), P (inorganic ion transport and metabolism; 6.72%), and category S (function unknown, 7.49%). Totally, 3,309 and 3,649 CDS could be annotated in GO (Supplementary Figure 2) and KEGG databases (Supplementary Figure 3), respectively. A total of 76 non-coding RNAs were also predicted, including 56 tRNA genes, 13 rRNA genes, and seven sRNA genes.

### Taxonomic identification of the strain CACMS001

**FIGURE 2:**
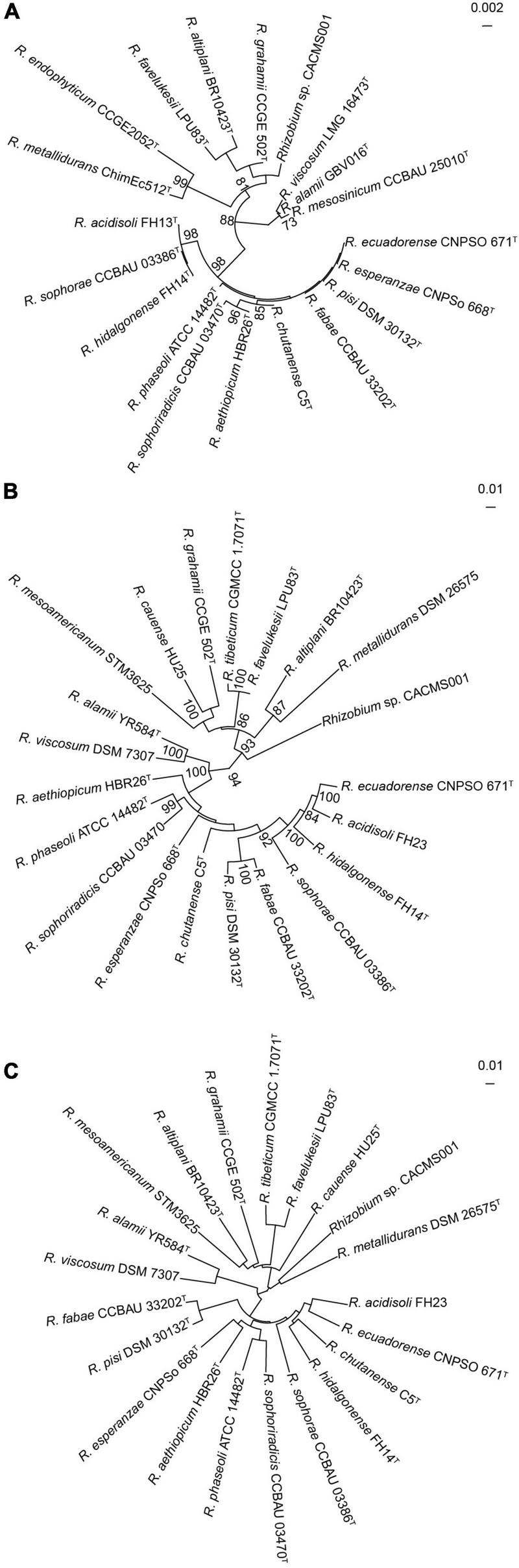
Taxonomic identification of strain CACMS001. **(A)** Maximum-likelihood phylogeny reconstructed from 16S rRNA gene. **(B)** Maximum-likelihood phylogeny reconstructed from concatenated *recA*, *atpD*, *glnII*, and *rpoB* genes. **(C)** Neighbor-joining phylogeny reconstructed from ANI.

Strain CACMS001 isolated from *P. umbellatus* was rod, aerobic, and identified as Gram-negative bacteria. Colonies were circular and pearl white on YMA medium at 28^°^C (Supplementary Figure 4). In the API 50 CHB test, strain CACMS001 could grow on the medium containing L-arabinose, ribose, D-xylose, galactose, glucose, fructose, mannose, rhamnose, esculin, raffinose, starch, glycogen, D-lyxose, D-tagatose, and D-fucose (Supplementary Table 5). These results were consistent with the typical carbon utilization features of *Rhizobium* (Kuykendall et al., 2015). The fatty acid profiles of strain CACMS001 were identified, and the principal cellular fatty acids were C_19:0_ cycloω8c and C_16:0_ (Supplementary Table 6), which are the characteristic compositions of rhizobia (Tighe et al., 2000). The identified 16S rRNA sequence also showed that strain CACMS001 belongs to the genus *Rhizobium*, and its most closed species was *R. grahamii* CCGE502 (98.92%, Supplementary Table 2), but these similarities were insufficient to classify strain CACMS001 into any species. Furthermore, the 16S rRNA gene tree also indicated that 16S rRNA gene sequences could not differentiate strain CACMS001 from its closely related *Rhizobium* species since the bootstrap support values for most nodes of were below 70% (Figure 2A). Based on the genomic annotation, the MLSA was carried out using four housekeeping genes, namely, *atpD*, *glnII*, *recA*, and *rpoB*. The strain CACMS001 showed 89.17–90.98% similarities with related *Rhizobium* species (Figure 2B and Supplementary Table 7).

The ANI and dDDH values were estimated from the genome sequences (Figure 2C). The ANIb values between strain CACMS001 and other *Rhizobium* species range from 76.80 to 82.25%, while the ANIb value between strain CACMS001 and *B. diazoefficiens* USDA 110 is 67.77% (Supplementary Table 7). The dDDH values between strain CACMS001 and other *Rhizobium* species range from 21.4 to 27.9%, while the dDDH value between strain CACMS001 and *B. diazoefficiens* USDA 110 is 17.9%.

### Effects of *Rhizobium* species on *P. umbellatus* and *A. gallica*

**FIGURE 3:**
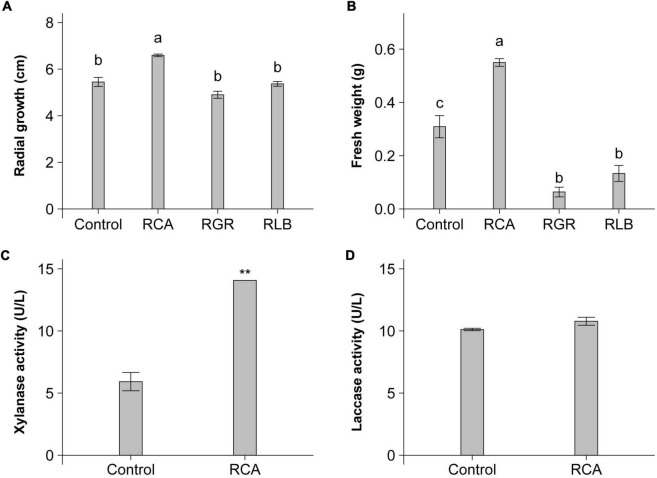
Influence of bacterial inoculant on *P. umbellatus* and *A. gallica*. **(A)** Influence on mycelial growth rate of *P. umbellatus*, **(B)** influence on fresh weight of *A. gallica*, **(C)** influence on extracellular xylanase activity of *A. gallica*, and **(D)** influence on extracellular laccase activity of *A. gallica.* RCA, *Rhizobium* sp. CACMS001; RGR, *R. grahamii* CCGE 502^T^; RLB, *R. leguminosarum* bv. *viciae* USDA 2370^T^. All data are presented as mean ± s.e. Bars with the same letter are not significantly different at the 5% LSD test. **p <0.01 between two groups, determined using two-sided Student’s *t* test.

We investigated the effect of strain CACMS001 on the growth of *P. umbellatus* and *A. gallica*, compared with *R. grahamii* CCGE502^T^ and *R. leguminosarum* bv. *viciae* USDA 2370^T^ that was the typical strain of *R. leguminosarum* bv. *viciae*, a well-known plant growth-promoting bacterium (PGPB). The effects of the *Rhizobium* species on mushroom growth were diverse, including positive, negative, and no effects. For *P. umbellatus*, only CACMS001 improved the growth of its mycelium with a 1.21-fold increase, compared with *P. umbellatus* cultivated alone (Figure 3A). For *A. gallica*, strain CACMS001 resulted in a 1.78-fold increase in fresh weight, while *R. grahamii* CCGE502^T^ and *R. leguminosarum* bv. *viciae* USDA 2370^T^ inhibited the growth of *A. gallica*, with a 0.21-fold and a 0.42-fold decrease, respectively, (Figure 3B).

Because of the obvious increase in the fresh weight of *A. gallica* co-cultured with strain CACMS001, we speculated that the activity of extracellular enzymes of *A. gallica* might be affected. As expected, the xylanase activity of *A. gallica* co-cultured with strain CACMS001 was 2.38-fold higher than that cultured alone (Figure 3C), while strain CACMS001 had no effect on the laccase activity of *A. gallica* mycelium (Figure 3D).

### Genome comparison of strain CACMS001, *R. grahamii*, and *R. metallidurans*

To investigate whether strain CACMS001 has unique genes related to fungal growth compared to other strains of *Rhizobium* species, comparative genomic analysis of strain CACMS001 with other closely related species (*R. grahamii* and *R. metallidurans*) was conducted. A total of 643 genes were identified specific to CACMS001, of which 168 genes could be clustered into 54 orthologous clusters. Based on KEGG annotation, these 168 genes were highly enriched in saccharide transporters (Supplementary Figure 5).

**FIGURE 4:**
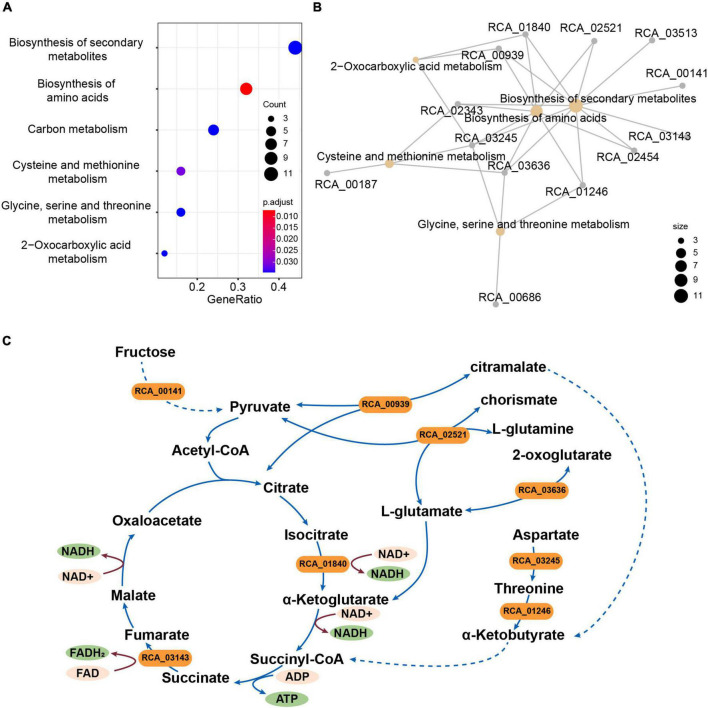
Genes under positive selection in strain CACMS001 were tightly linked to energy metabolism. **(A)** Enriched pathways in genes under positive selection, **(B)** map of genes enriched in the KEGG pathway, and **(C)** genes in enrichment pathways participating in energy metabolism. Solid lines represent one-step reactions; dashed lines represent multi-step reactions; arrows represent the directions of reactions; and rounded rectangle represent genes.

A total of 2,539 single-copy genes from the strains CACMS001, *R. grahamii*, and *R. metallidurans* genomes were scanned to identify genes under selection. We identified 41 genes that appeared to be under positive selection (Supplementary Table 8). These genes were predicted to be related to the biosynthesis of secondary metabolites; biosynthesis of amino acids; carbon metabolism; cysteine and methionine metabolism; glycine, serine, and threonine metabolism; and 2-oxocarboxylic acid metabolism based on KEGG annotation (Figures 4A,B). We found that eight genes (RCA_00939, RCA_01246, RCA_01840, RCA_02343, RCA_02454, RCA_02521, RCA_03245, and RCA_03636) were involved in the tricarboxylic acid (TCA) cycle (Supplementary Table 8 and Figure 4C). We also found three other genes under positive selection related to the TCA cycle, namely, RCA_00141, RCA_01246, and RCA_03143.

### Nitrogen fixation and nodulation genetic repertoire

**FIGURE 5:**
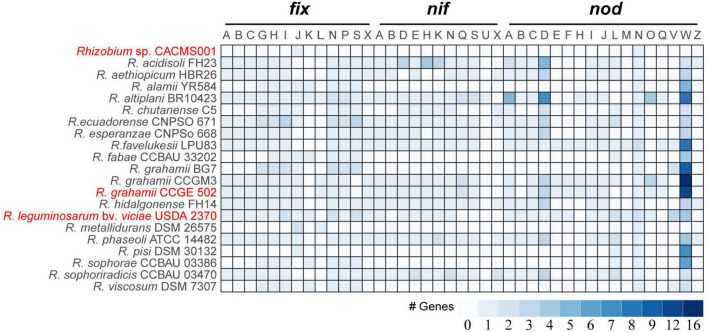
*nif*, *fix*, and *nod* genes in *Rhizobium* species. Red words represent species whose effect on *P. umbellatus* and *A. gallica* have been evaluated.

It is well known that *fix*, *nif*, and *nod* genes are regarded as key factors in rhizobia-legume symbiotic systems. Among 21 species, strain CACMS001 had the smallest genetic repertoire for nitrogen fixation and nodulation, with one *fixJ* and one *nodN* (Figure 5). *R. metallidurans* DSM 26575 remains two *fixJ*s, one *fixL*, and one *nodN*. *R. grahamii* BG7 possessed seven *fix* genes (*fixG*, *fixH*, *fixI*, *fixJ*, *fixN*, *fixP*, and *fixS*) and two *nod* genes (*nodV* and *nodW*). Strain CACMS001, *R. metallidurans* DSM 26575, *R. grahamii*, and strain BG7 lost *nif* genes. However, *R. grahamii* CCGE 502 and *R. grahamii* CCGM3 possessed *nif* genes.

### Lignocellulose degradation enzymes

**FIGURE 6:**
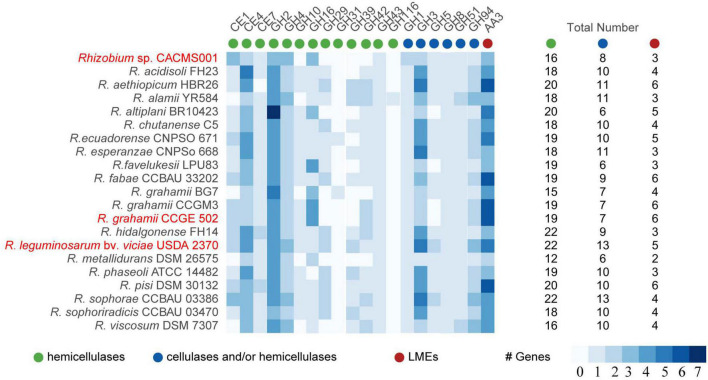
Lignocellulose degradation enzyme coding genes in *Rhizobium* species. Red words represent species whose effects on *P. umbellatus* and *A. gallica* have been evaluated.

Lignin-modifying enzymes, cellulases, and hemicellulases are responsible for lignocellulose degradation. According to the classification of CAZymes, these lignocellulose degradation enzymes can be classified into auxiliary activities (AA), carbohydrate esterase (CE), and glycoside hydrolase (GH; Bredon, 2018). Totally, 16 hemicellulases, eight cellulases, and/or hemicellulases, and one LME were identified in strain CACMS001 (Figure 6). Among all strains, *R. leguminosarum* bv. *viciae* USDA 2370 has the most genes involved in lignocellulose degradation, while *Rhizobium metallidurans* DSM 26575 has the fewest. There were 13 families (CE1, CE4, CE7, GH16, GH43, GH1, GH3, GH5, GH5, GH8, GH51, GH94, and AA3) involved in lignocellulose degradation in both CACMS001 and *R. leguminosarum* bv. *viciae* USDA 2370, and the number of genes classified into these families has been shown in Figure 6.

### Antimicrobial peptides

**FIGURE 7:**
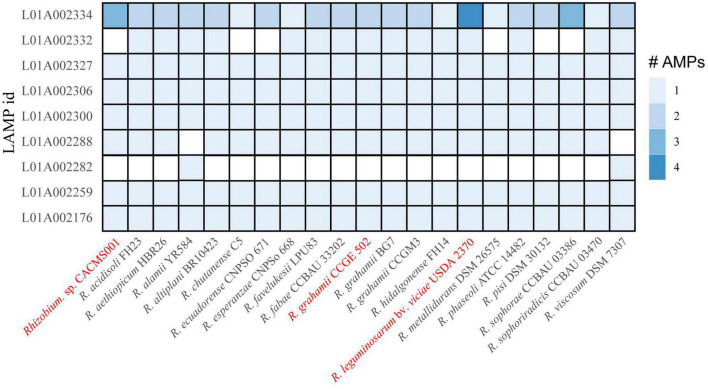
AMP in *Rhizobium* species. Red words represent species whose effects on *P. umbellatus* and *A. gallica* have been evaluated.

Genes encoding eight AMPs were identified in *Rhizobium* species in this study. All the eight AMPs were originally isolated from *Fusarium culmorum* and have antifungal activity (Bunkers et al., 2003). The strain CACMS001 had most AMP coding genes, among which L01A002334 coding gene has three copies (Figure 7).

### Secondary metabolite gene clusters

**FIGURE 8:**
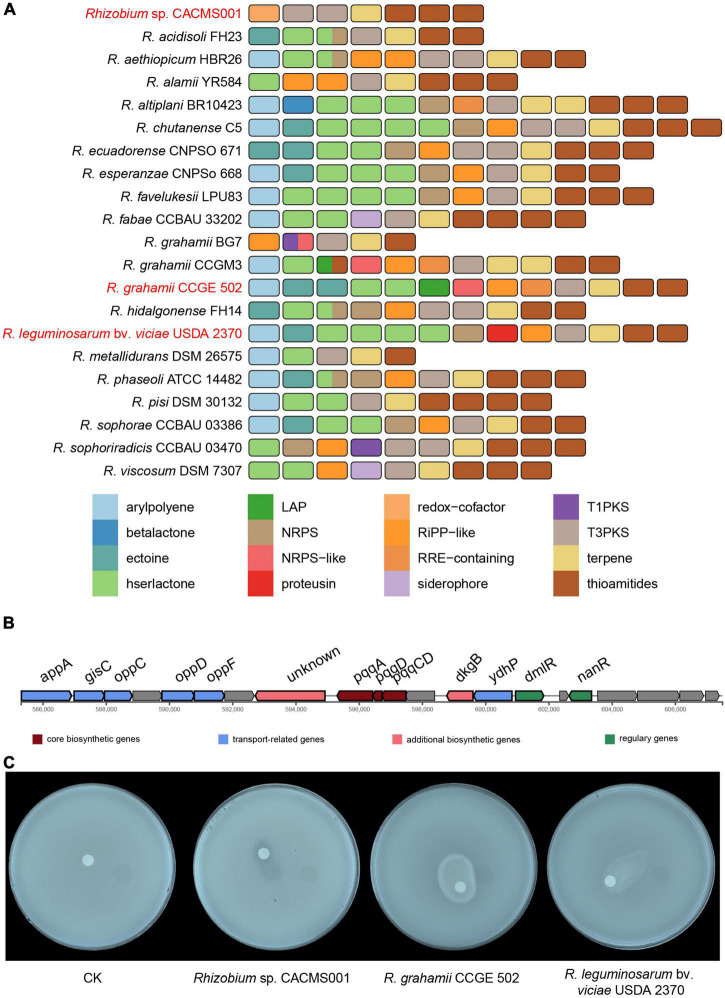
Comparison of secondary metabolite biosynthetic gene clusters in *Rhizobium* species. **(A)** Comparison of biosynthetic gene clusters between strain CACMS001 and other closely related species. **(B)** PQQ biosynthetic gene cluster in strain CACMS001. **(C)** Phosphate solubilizing by the strain CACMS001 on Pikovskaya medium.

Totally, seven secondary metabolites gene clusters were identified in the genome of strain CACMS001, including one terpene, one redox cofactor, two type III PKS (T3PKs), and three thioamides (Figure 8A). Two of these gene clusters located on the chromosome and other five clusters located on the plastid. Two other *Rhizobium* strains evaluated on *P. umbellatus* showed the similar secondary metabolite profile (Figure 8A). They both have 13 secondary metabolites gene clusters, including arylpolyene, ectoine, hserlactone, non-ribosomal peptide synthetase (NRPS), ribosomally synthesized and post-translationally modified peptides-like (RiPP-like), thioamides, and T3PKs. Compared to *R. grahamii* CCGE 502^T^ and *R. leguminosarum* bv. *viciae* USDA 2370^T^, strain CACMS001 specially acquired one redox cofactor cluster but lost other clusters including arylpolyene, ectoine, hserlactone, NRPS, and RiPP-like clusters. Overall, four genes in the redox cofactor cluster in strain CACMS001 encode *pqqE*, *pqqD*, *pqqC*, and *pqqB* (Figure 8B), and all of them involved in the synthesis of pyrroloquinoline quinone (PQQ), which is a peptide-derived redox cofactor for the assembly of glucose dehydrogenase (GDH; Zhu and Klinman, 2020). In addition, neither IAA biosynthetic gene nor AcdS-coding gene was found in strain CACMS001 genome. Since GDH-dependent phosphate solubilization has been observed in numerous bacteria, we also tested the phosphate solubilization activity and confirmed that strain CACMS001 could solubilize phosphate (Figure 8C).

### Expression of phosphate-related genes in strain CACMS001

**FIGURE 9:**
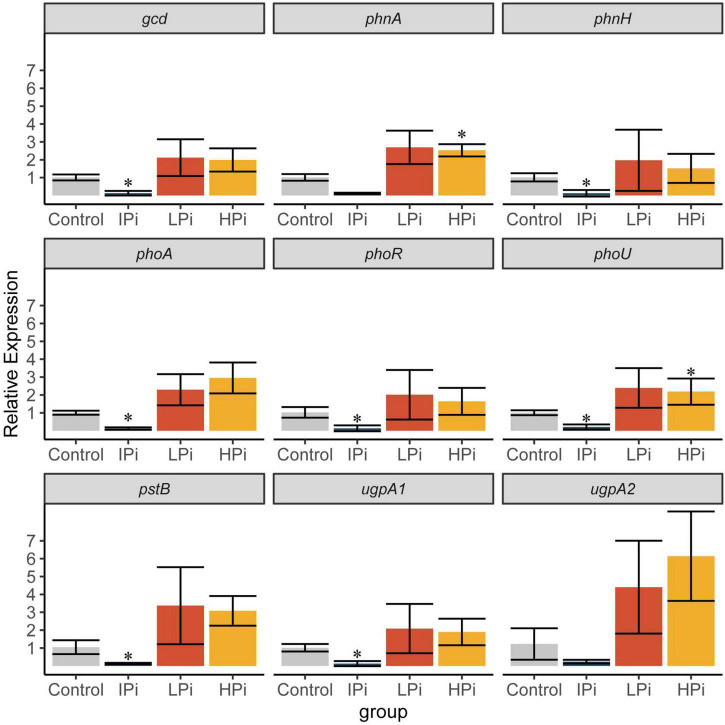
Expression of phosphate-related genes. Mean ± SD values of 2^–ΔΔCt^ are shown (*n* = 3). IPi, YMB with 10 g⋅L^–1^ of Ca_3_ (PO_4_)_2_; LPi, YMB with 5 mmol⋅L^–1^ of K_2_HPO_4_; HPi, YMB with 10 mmol⋅L^–1^ of K_2_HPO_4_. Control was YMB. *P < 0.05 compared with the control group.

To investigate the mechanisms by which strain CACMS001 solubilized the phosphate, the expression of phosphate-related genes in different pathways was detected by qPCR. Compared to the control group, *phnA* and *phoU* were upregulated in the HPi group, and *gcd*, *phnH*, *phoA*, *phoR*, *phoU*, *pstB*, and *ugpA1* were downregulated in the IPi group (Figure 9). Since *phnA* is involved in organic P mineralization and *phoR* is involved in P regulation, this result indicated that organic P mineralization and P regulation were upregulated when strain CACMS001 grows in the phosphate-rich environment. Interestingly, four pathways involved in phosphate solubilization, including inorganic P solubilization, organic P mineralization, P regulation, and P transportation, were downregulated when insoluble phosphate, instead of soluble phosphate, was present in the environment.

## Discussion

To our knowledge, CACMS001 was the first *Rhizobium* strain isolated from fungi according to MLSA and genomic analysis results. The strain CACMS001 is a putative new species because the similarities less than 96% in MLSA (Yan et al., 2017) have been observed between strain CACMS001 and the described *Rhizobium* species; the dDDH similarities of strain CACMS001 and closely related species were lower than 70% (Meier-Kolthoff et al., 2014), and the highest ANIb (82.25%) between strain CACMS001 and other *Rhizobium* species was less than 95% (Goris et al., 2007).

The effects of strain CACMS001 on fungi growth were further evaluated by co-culturing CACMS001 with *P. umbellatus* and *A. gallica* because the cultivation of *P. umbellatus* relies on *A. gallica* (Xing et al., 2013). Previously reported MGPB strains could increase mycelia growth by 29 to 180% (Suarez et al., 2020; Kumari and Naraian, 2021; Sari and Aryantha, 2021). The promoting rates of CACMS001 on *P. umbellatus* and *A. gallica* were 21 and 78%, respectively. The extracellular xylanase activity of *A. gallica* was increasing more than 2-fold when co-cultured with strain CACMS001 and may also contribute to the mycelia growth of *A. gallica*. Conversely, no effect was observed in *R. grahamii* CCGE502^T^ (a taxonomically related strain) or *R. leguminosarum* bv. *viciae* USDA 2370^T^ (a typical PGPB) on the growth of *P. umbellatus* and *A. gallica*. The result confirmed that CACMS001 was a potential MGPB and could have unique features compared to other *Rhizobium* species.

To further elucidate the mechanism by which CAMCS001 promotes the growth of *P. umbellatus* and *A. gallica*, we first compared the genome characteristics of strain CACMS001 with those of the closely related species. Compared with other *Rhizobium* strains, CACMS001 has unique genomic characteristics, which can utilize nutrients more efficiently. Enriched genes for saccharide transporters in specific gene clusters showed that CACMS001 has the ability to transport carbon sources. Furthermore, genes under positive selection related to the TCA cycle indicated that CACMS001 could have more efficiently carbon metabolism (Varela et al., 2019).

Lignocellulose is composed of cellulose, lignin, and hemicellulose and is the main component of the mushroom substrate for agricultural and/or agro-industrial by-products, such as straw, grass, sawdust, and coffee pulp (Vieira et al., 2019). AA1 and AA2 are two CAZyme gene families crucial for lignin degradation (Jiménez et al., 2014) and not found in the genome of CACMS001 and other *Rhizobium* species CACMS001 has lower gene numbers of cellulases and hemicellulases than *R. grahamii* CCGE502^T^ and *R. leguminosarum* bv. *viciae* USDA 2370^T^, suggesting that lignin degradation is not important for strain CACMS001 to promote fungal growth.

We further investigate whether CACMS001 could fix nitrogen. Interestingly, almost all nitrogen-fixation genes have been lost in CACMS001 genome. *Fix* gene (*fixJL*, *fixNOQP*, *fixGHIS*, and *fixABCX*) products are involved in symbiosis-specific respiration, electron transfer to the nitrogenase, or oxygen regulation of nitrogen fixation (Tian, 2011). Among them, *fixJL* was the most important factor and interacts with other *fix* genes (De Meyer et al., 2016; Roy et al., 2020), but *fixJL* lost in CACMS001. Totally, seven *nif* genes (*nifA*, *nifB*, *nifD*, *nifE*, *nifH*, *nifK*, and *nifN*) play crucial roles in nitrogen fixation (Tian, 2011) and were identified in *R. grahamii* CCGE502^T^ and *R. leguminosarum* bv. *viciae* USDA 2370^T^, suggesting *R. grahamii* CCGE502^T^ and *R. leguminosarum* bv. *viciae* USDA 2370^T^ are nitrogen-fixing bacteria, which is consistent with previous studies (Saïdi et al., 2013). However, the nitrogen-fixing *Rhizobium* species showed no effects on fungal growth, and none of *nif* genes detected in strain CACMS001 reveals that nitrogen fixation may be insufficient to stimulate *P. umbellatus* and *A. gallica* growth.

Mushroom growth-promoting bacterias belonging to *Bacillus* and *Pseudomonas* have been shown to produce IAA (Suarez et al., 2020), and several bacteria are reported to generate AcdS for reducing ethylene to relieve fungal growth inhibition (Chen et al., 2013). Neither IAA biosynthetic gene nor AcdS coding gene was identified in CACMS001 genome, suggesting that strain CACMS001 promotes fungal growth unregulated by auxin or ethylene.

Antibiotic biosynthetic clusters in the CACMS001 genome were further investigated, and none of them was identified. We then investigated AMPs, the small peptides with length ranging from 12 to 100 amino acid residues, which have a broad spectrum of target organisms including fungi (Huan et al., 2020). Although CACMS001 had most AMP-coding genes, there was no significant difference in the number and types of AMP coding genes in the CACMS001 genome, compared to other *Rhizobium* species. The loss of antibiotic biosynthetic clusters and specific AMPs suggests that strain CACMS001 may not produce antibiotics or AMPs to affect fungal growth.

However, a species-specific PQQ biosynthetic cluster was present in the CACMS001 genome. PQQ is a peptide-derived redox cofactor for the assembly of the GDH holoenzyme produced by prokaryotes, which acts in the oxidation of glucose to gluconic acid (Zhu and Klinman, 2020), and GDH-dependent phosphate solubilization has been detected in bacteria (Ran et al., 2016). The PQQ biosynthetic cluster in CACMS001 genome suggested CACMS001 could be anticipated to have the ability to solubilize insoluble phosphates, which were supported by the halo zone observed on PVK medium and the gene copy number of the genes encoding phosphate-related enzymes in both P-deficient and P-sufficient environment. By contrast, almost all genes were downregulated when strain CACMS001 was exposed to insoluble phosphate, indicating CACMS001 may be specifically regulated by insoluble phosphate, which was also indirectly demonstrated that only the halo zone but not strain growth was observed on PVK medium. However, the mechanism by which inorganic phosphate and nutrients crosstalk is associated with bacterial growth is just beginning to be understood (Santos-Beneit, 2015). A work performed in a *R. leguminosarum* strain showed that phosphate-rich conditions downregulate *pssA*, a gene involved in exopolysaccharide biosynthesis in both wild-type strains and the *phoB* mutants (Janczarek and Urbanik-Sypniewska, 2013), which is consistent with our results. These results suggest that the effects of inorganic phosphate-rich conditions on *Rhizobium* growth are complex and warrant further exploration.

## Conclusion

In conclusion, strain CACMS001 was isolated from *P. umbellatus* sclerotia for the first time and was confirmed to be an MGPB belonging to *Rhizobium* with the ability to promote mycelial growth. Strain CACMS001 is significantly different from other *Rhizobium* species and is a potential new species. Based on our results, strain CACMS001 ought to have more efficiently carbon metabolism and play a phosphate solubilization role in its mutualistic relationship with its fungal host, and in turn, its fungal host may provide carbon for strain CACMS001. We believe this novel bacterium is of great practical value because of its positive effects on both *P. umbellatum* and *A. gallica*.

## Data Availability Statement

The datasets presented in this study can be found in online repositories. The names of the repository/repositories and accession number(s) can be found in the article/Supplementary Material.

## Author contributions

ZH, TL, PH, YZ, and JZ carried out the experimentation of this work. YZ and JZ collected and identified strains for this research. TL and PH performed the co-culture experiments. ZH performed bioinformatics analysis. ZH, LH, and YY wrote the manuscript. All authors contributed to the article and approved the submitted version.

## Conflict of Interest

The authors declare that the research was conducted in the absence of any commercial or financial relationships that could be construed as a potential conflict of interest.

## Publisher’s Note

All claims expressed in this article are solely those of the authors and do not necessarily represent those of their affiliated organizations, or those of the publisher, the editors and the reviewers. Any product that may be evaluated in this article, or claim that may be made by its manufacturer, is not guaranteed or endorsed by the publisher.

## Footnotes

1
http://www.geneontology.org/

2
http://www.genome.jp/kegg/pathway.html
