# Supplementary material for: Isolation, genomic characterization, and mushroom growth-promoting effect of the first fungus-derived Rhizobium
Source: Front Microbiol. 2022 Jul 22;13:947687. doi: 10.3389/fmicb.2022.947687 (PMC9354803; doi:10.3389/fmicb.2022.947687)
Supplement: Supplementary file 1 [file Data_Sheet_1.docx]

**Table S1**. The sequence used in *fix*. *nif* and *nod* gene search

| Class | Name | Organism ID | Uniprot ID | Genebank ID |
| --- | --- | --- | --- | --- |
| fix | fixA | *R.* *leguminosarum* bv. *viciae* (strain 3841) | Q1M7V4 | CAK10424.1 |
|  | fixB | *R.* *leguminosarum* bv. *viciae* (strain 3841) | Q1M7V5 | CAK10423.1 |
|  | fixC | *R.* *leguminosarum* bv. *viciae* (strain 3841) | Q1M7V6 | CAK10422.1 |
|  | fixG | *R.* *leguminosarum* bv. *viciae* (strain 3841) | Q1M7U6 | CAK10433.1 |
|  | fixH | *R.* *leguminosarum* bv. *viciae* (strain 3841) | Q1M7U5 | CAK10434.1 |
|  | fixI | *R.* *leguminosarum* bv. *viciae* (strain 3841) | Q1M7U4 | CAK10435.1 |
|  | fixJ | *E. meliloti* (strain1021) | P10958 | CAA79898.1 |
|  | fixK | B. *japonicum* USDA 110 | P29286 | BAC51326.1 |
|  | fixL | *E. meliloti* (strain1021) | P10955 | CAA79897.1 |
|  | fixN | *R.* *leguminosarum* bv. *viciae* (strain 3841) | Q1M7V0 | CAK10429.1 |
|  | fixO | *R.* *leguminosarum* bv. *viciae* (strain 3841) | Q1M7U9 | CAK10430.1 |
|  | fixP | *R.* *leguminosarum* bv. *viciae* (strain 3841) | Q1M7U7 | CAK10432.1 |
|  | fixQ | *R.* *leguminosarum* bv. *viciae* (strain 3841) | Q1M7U8 | CAK10431.1 |
|  | fixS | *R.* *leguminosarum* bv. *viciae* (strain 3841) | Q1M7U3 | CAK10436.1 |
|  | fixX | *R.* *leguminosarum* bv. *viciae* (strain 3841) | Q1M7V7 | CAK10421.1 |
| nif | nifA | *R.* *leguminosarum* bv. *viciae* (strain 3841) | Q1M7V8 | CAK10420.1 |
|  | nifB | *R.* *leguminosarum* bv. *viciae* (strain 3841) | Q1M7V9 | CAK10419.1 |
|  | nifD | *R.* *leguminosarum* bv. *viciae* (strain 3841) | Q1M7Z3 | CAK10384.1 |
|  | nifE | *R.* *leguminosarum* bv. *viciae* (strain 3841) | Q1M7Z5 | CAK10382.1 |
|  | nifF | *K. pneumoniae* | P04668 | CAA31680.1 |
|  | nifH | *R.* *leguminosarum* bv. *viciae* (strain 3841) | Q1M7Z2 | CAK10385.1 |
|  | nifJ | *K. pneumoniae* | P03833 | CAA31665.1 |
|  | nifK | *R.* *leguminosarum* bv. *viciae* (strain 3841) | Q1M7Z4 | CAK10383.1 |
|  | nifL | *K. pneumoniae* | P06772 | CAA31681.1 |
|  | nifM | *K. pneumoniae* | P0A3Y9 | CAA31679.1 |
|  | nifN | *R.* *leguminosarum* bv. *viciae* (strain 3841) | Q1M7Z6 | CAK10381.1 |
|  | nifQ | *K. pneumoniae* | P10392 | CAA31684.1 |
|  | nifS | *K. pneumoniae* | P05344 | CAA31118.1 |
|  | nifU | *K. pneumoniae* | P05343 | CAA31117.1 |
|  | nifV | *K. pneumoniae* | P05345 | CAA31119.1 |
|  | nifW | *K. pneumoniae* | P09137 | CAA31120.1 |
|  | nifX | *K. pneumoniae* | P09136 | CAA31116.1 |
|  | nifY | *K. pneumoniae* | P09135 | CAA31114.1 |
| Nod | nodA | *R.* *leguminosarum* bv. *viciae* (strain 3841) | Q1M7W9 | CAK10409.1 |
|  | nodB | *R.* *leguminosarum* bv. *viciae* (strain 3841) | Q1M7W8 | CAK10410.1 |
|  | nodC | *R.* *leguminosarum* bv. *viciae* (strain 3841) | Q1M7W7 | CAK10411.1 |
|  | nodD | *R.* *leguminosarum* bv. *viciae* (strain 3841) | Q1M7X0 | CAK10408.1 |
|  | nodE | *R.* *leguminosarum* bv. *viciae* (strain 3841) | Q1M7X2 | CAK10406.1 |
|  | nodF | *R.* *leguminosarum* bv. *viciae* (strain 3841) | Q1M7X1 | CAK10407.1 |
|  | nodH | *R. tropici* | P52994 | CAA60912.1 |
|  | nodI | *R.* *leguminosarum* bv. *viciae* (strain 3841) | Q1M7W6 | CAK10412.1 |
|  | nodJ | *R.* *leguminosarum* bv. *viciae* (strain 3841) | Q1M7W5 | CAK10413.1 |
|  | nodL | *R.* *leguminosarum* bv. *viciae* (strain 3841) | Q1M7X3 | CAK10405.1 |
|  | nodM | *R.* *leguminosarum* bv. *viciae* (strain 3841) | Q1M7X4 | CAK10404.1 |
|  | nodN | *R.* *leguminosarum* bv. *viciae* (strain 3841) | Q1M7X5 | CAK10403.1 |
|  | nodO | *R.* *leguminosarum* bv. *viciae* (strain 3841) | Q1M7X8 | CAK10399.1 |
|  | nodP | *R. tropici* | P52995 | CAA60913.1 |
|  | nodQ | *R. tropici* | P52978 | CAA60914.1 |
|  | nodS | *R. tropici* | Q53514 | AAB34510.1 |
|  | nodU | *R. tropici* | Q53515 | AAB34511.1 |
|  | nodV | *B. japonicum* (USDA110) | P15939 | AAA26231.1 |
|  | nodW | *B. japonicum* (USDA110） | P15940 | AAA26232.1 |
|  | nodX | *R.* *leguminosarum* bv. *viciae* | P08888 | CAA30799.1 |
|  | nodZ | *R. phaseoli* | A0A192TJI1 | ANL94693.1 |

**Table S2**. The 16S sequences used in phylogenetic analysis

| Species | Strain | Accession number | Sequence similarities (%) with  *Rhizobium* sp. CACMS001 |
| --- | --- | --- | --- |
| *R. acidisoli* | FH13 | KJ921033 | 97.93% |
| *R. aethiopicum* | HBR26 | jgi.1052919 | 97.76% |
| *R. alamii* | GBV016 | AM931436 | 98.04% |
| *R. altiplani* | BR10423 | LNCD01000038 | 98.19% |
| *R. cauense* | CCBAU 101002 | JQ308326.1 | 97.47% |
| *R. chutanense* | C5 | KJ438829 | 98.41% |
| *R. ecuadorense* | CNPSO 671 | LFIO01000095 | 97.98% |
| *R. endophyticum* | CCGE2052 | EU867317 | 97.82% |
| *R. esperanzae* | CNPSo 668 | KC293513 | 98.71% |
| *R. fabae* | CCBAU 33202 | DQ835306 | 98.45% |
| *R. favelukesii* | LPU83 | HG916852 | 98.19% |
| *R. grahamii* | CCGE 502 | AEYE01000061 | 98.92% |
| *R. hidalgonense* | FH14 | KJ921034 | 97.93% |
| *R. mesoamericanum* | CCGE 501 | JF424606.1 | 97.09% |
| *R. mesosinicum* | CCBAU 25010 | DQ100063 | 97.85% |
| *R. metallidurans* | ChimEc512 | JX678769 | 97.76% |
| *R. phaseoli* | ATCC 14482 | EF141340 | 97.98% |
| *R. pisi* | DSM 30132 | RJJT01000050 | 97.98% |
| *R. sophorae* | CCBAU 03386 | KJ831229 | 97.95% |
| *R. sophoriradicis* | CCBAU 03470 | RQIH01000042 | 97.76% |
| *R. tibeticum* | CGMCC 1.7071 | EU256404.1 | 98.60% |
| *R. viscosum* | LMG 16473 | AJ639832 | 97.75% |
| *B. diazoefficiens* | USDA 110 | D13430.1 | 88.66% |

**Table S3**. The genomes used in present study

| Species | Strain | Genome Accession |
| --- | --- | --- |
| *R. acidisoli* | FH23 | GCF_002531755.2 |
| *R. aethiopicum* | HBR26 | GCF_900094625.1 |
| *R. alamii* | YR584 | GCF_000799895.1 |
| *R. altiplani* | BR10423 | GCF_001542405.1 |
| *R. cauense* | HU25 | GCF_019511525.1 |
| *R. chutanense* | C5 | GCF_002531935.1 |
| *R. ecuadorense* | CNPSO 671 | GCF_001187535.1 |
| *R. esperanzae* | CNPSo 668 | GCF_002204185.1 |
| *R. fabae* | CCBAU 33202 | GCF_003985135.1 |
| *R. favelukesii* | LPU83 | GCF_000577275.2 |
| *R. grahamii* | CCGE 502 | GCF_000298315.2 |
| *R. grahamii* | BG7 | GCF_009498215.1 |
| *R. grahamii* | CCGM3 | GCF_003351175.1 |
| *R. hidalgonense* | FH14 | GCF_002531855.1 |
| *R. leguminosarum* bv. *viciae* | USDA 2370 | GCF_002008365.1 |
| *R. mesoamericanum* | STM3625 | GCF_000312665.1 |
| *R. metallidurans* | DSM 26575 | GCF_014196505.1 |
| *R. phaseoli* | ATCC 14482 | GCF_003985125.1 |
| *R. pisi* | DSM 30132 | GCF_003938655.1 |
| *R. sophorae* | CCBAU 03386 | GCF_013087515.1 |
| *R. sophoriradicis* | CCBAU 03470 | GCF_003939025.1 |
| *R. tibeticum* | CGMCC 1.7071 | GCF_900110205.1 |
| *R. viscosum* | DSM 7307 | GCF_014873945.1 |
| *B. diazoefficiens* | USDA 110 | GCF_001642675.1 |

**Table S4** Primers used in qPCR in present study

| Gene | Forward primer | Reverse primer |
| --- | --- | --- |
| *rrn16(RCA_02838)* | TGATGAAGGCCTTAGGGTTG | TACGCCCAGTAAATCCGAAC |
| *phoR* (RCA_00175) | GTTTCATCGAGACCATCCAG | GGTCTGATCGAACATGATGC |
| *pstB* (RCA_00179) | ATGAACGACACGATCGACAG | TAGATCGTCTTCGGGAATGG |
| *phoU* (RCA_00180) | TTGAGCATCTCTCCGAACTG | TACATCGCGTCGATTTCCTC |
| *gcd* (RCA_00770) | CTGGCAAGGAAAAGTGGAAG | TGTCGTGGAAATAGGTGACG |
| *phoA* (RCA_01833) | ACATCGAAAAGGAGCCTGTC | GAAGAAGCCCTTCTCATTCG |
| *phnA* (RCA_02149) | TTGAAGGTCAAGGGCACATC | CGAACTCCGTTTTCAACACC |
| *phnH* (RCA_03842) | AAGACCGAAGCCCACAATAC | TCCATCATCCGCTTGAAGAC |
| *ugpA1* (RCA_03960) | TGCTGTGGTGGTTCATGTTC | TAAGCCGATGATCCCAGTTG |
| *ugpA2* (RCA_04824) | TGACCGTGGTGTTCTTCTTC | CCTGCAGCGAATTGAGATAG |

**Table S5** The phenotypic characteristics of strain CACMS001

| API | Results |
| --- | --- |
| Control | - |
| glycerinum | - |
| Erythrose | - |
| D-arabinose | - |
| L-arabinose | + |
| Ribose | W |
| D-xylose | W |
| L-xylose | - |
| Adonitol | - |
| β-methyl-D-xyloside | - |
| Galactose | + |
| Glucose | + |
| Furctose | W |
| Mannose | + |
| Sorbose | - |
| Rhamnose | W |
| Dulcitol | - |
| Inositol | - |
| Mannitol | - |
| Sorbitol | - |
| α-metyl-D-mannosidase | - |
| α-metyl-D-glucoside | - |
| N-Acetylglucosamine | - |
| Amygdalin | - |
| Arbutin | - |
| Esculin | + |
| Salicoside | - |
| Cellobiose | - |
| Maltose | - |
| Lactose | - |
| Melibiose | - |
| Sucrose | - |
| Trehalose | - |
| Inulin | - |
| Melezitose | - |
| Raffinose | W |
| Starch | W |
| Glycogen | W |
| Xylitol | - |
| Geranyl | - |
| D-Turanose | - |
| D-Lyxose | W |
| D-Tagatose | W |
| D-Fucose | + |
| L-Fucose | - |
| D-Arabitol | - |
| L-Arabitol | - |
| Gluconat | - |
| 2-keto-gluconate | - |
| 5-keto-gluconate | - |

Note: + growth or resistant; ‒ no growth or sensitive; w weakly positive

**Table S6** Cellular fatty acid contents of strain CACMS001 and the type strains of other related *Rhizobium* species.

| **Fatty Acid** | *Rhizobium* sp. CACMS001 | *R. grahamii* CCGE 502^T^ | *R. metallidurans* ChimEc512^T^ |
| --- | --- | --- | --- |
| C_16:0_ | 18.37 | 5.75 | 5.60 |
| C_16:0_ 3-OH | 2.02 | 4.31 | ND |
| C_17:0_ cyclo | 3.02 | 1.21 | ND |
| C_18:0_ | 3.64 | 5.24 | 5.60 |
| C_18:0_ 3-OH | 1.7 | 5.98 | 2.1 |
| C_18:1_ω7c | ND | 36.42 | 67.40 |
| 11-Methyl C_18:1_ω7c | 4.66 | 0.45 | 6.7 |
| C_19:0_ cycloω8c | 41.42 | 21.64 | ND |
| Summed Feature 2 * | 8.11 | ND | ND |
| Summed Feature 3 * | 1.27 | ND | ND |
| Summed Feature 8 * | 12.76 | ND | ND |
| Summed Feature 2** | ND | 16.66 | 10.4 |

Note: Values are percentages of total fatty acids. Fatty acids representing < 1% in all strains were omitted. ND, Not detected. The *Rhizobium* sp. CACMS001 data were obtained in present study. The related species data were obtained from previous literature (Lopez-Lopez et al., 2012, Grison et al., 2015).

* Summed features consist of two or more fatty acids that could not be separated by the Microbial Identification System detected in present study. Summed feature 2 comprised aldehyde-C_12:0_ and/or unknown equivalent chain length (ECL) 10.9525; summed feature 3 comprised C_16:1_ω7c and/or C_16:1_ω6c; summed feature 8 comprised C_18:1_ω7c and/or C_18:1_ω6c

** Summed features consist of two or more fatty acids that could not be separated by the Microbial Identification System detected in previous study. Summed feature 2** contained one or more of C_12:0_ aldehyde, unknown ECL 10.928, iso-C_16:1_ I and C_14:0_ 3-OH

**Table S7** Sequence similarities and ANI of the *Rhizobium* sp. CACMS001 and related type strains in genus *Rhizobium*

| Strains | Sequence similarities (%) with  *Rhizobium* sp. CACMS001 | | | | |  | | ANI (%) | | dDDH (%) |
| --- | --- | --- | --- | --- | --- | --- | --- | --- | --- | --- |
|  | *atpD* | *glnII* | *recA* | *rpoB* | MLSA |  | ANIb | | ANIm |  |
| *R. acidisoli* FH23 | 89.98 | 88.66 | 87.00 | 90.92 | 89.89 |  | 77.60 | | 84.61 | 23.1 |
| *R. aethiopicum* HBR26^T^ | 90.55 | 88.95 | 86.54 | 90.63 | 89.81 |  | 77.27 | | 84.35 | 22.6 |
| *R. alamii* YR584 | 89.50 | 88.63 | 87.24 | 90.77 | 89.77 |  | 76.80 | | 84.24 | 22.1 |
| *R. altiplani* BR10423^T^ | 91.08 | 85.69 | 88.02 | 89.68 | 89.17 |  | 77.89 | | 84.78 | 23.2 |
| *R. cauense* HU25 | 89.80 | 90.01 | 88.12 | 91.06 | 90.27 |  | 77.56 | | 84.90 | 22.9 |
| *R. chutanense* C5^T^ | 93.31 | 84.73 | 88.49 | 92.34 | 90.96 |  | 77.68 | | 84.65 | 23.0 |
| *R. ecuadorense* CNPSO 671^T^ | 89.21 | 89.91 | 87.00 | 90.60 | 89.75 |  | 77.61 | | 84.64 | 23.1 |
| *R. esperanzae* CNPSo 668^T^ | 90.34 | 89.05 | 87.00 | 90.29 | 89.67 |  | 77.32 | | 84.45 | 22.8 |
| *R. fabae* CCBAU 33202^T^ | 89.99 | 88.95 | 87.47 | 90.68 | 89.86 |  | 77.47 | | 84.54 | 22.8 |
| *R. favelukesii* LPU83^T^ | 91.44 | 86.07 | 88.36 | 92.44 | 90.82 |  | 77.90 | | 84.78 | 21.4 |
| *R. grahamii* CCGE 502^T^ | 93.10 | 83.96 | 87.93 | 92.05 | 90.58 |  | 77.62 | | 84.89 | 23.1 |
| *R. hidalgonense* FH14^T^ | 90.05 | 88.95 | 86.82 | 90.41 | 89.64 |  | 77.31 | | 84.50 | 22.7 |
| *R. mesoamericanum* STM3625 | 93.03 | 87.70 | 93.83 | 90.38 | 90.98 |  | 77.28 | | 84.46 | 22.5 |
| *R. metallidurans* DSM 26575 | 88.54 | 85.59 | 87.37 | 91.59 | 89.62 |  | 82.25 | | 86.82 | 27.9 |
| *R. phaseoli* ATCC 14482^T^ | 90.06 | 88.86 | 87.65 | 90.51 | 89.80 |  | 77.49 | | 84.55 | 22.9 |
| *R. pisi* DSM 30132^T^ | 89.22 | 88.66 | 87.47 | 90.22 | 89.44 |  | 77.45 | | 84.54 | 22.8 |
| *R. sophorae* CCBAU 03386^T^ | 88.66 | 89.05 | 87.74 | 90.29 | 89.46 |  | 77.51 | | 84.44 | 23.0 |
| *R. sophoriradicis* CCBAU 03470^T^ | 89.71 | 88.76 | 87.00 | 90.63 | 89.70 |  | 77.45 | | 84.51 | 22.8 |
| *R. tibeticum* CGMCC 1.7071^T^ | 91.03 | 86.46 | 88.55 | 92.15 | 90.67 |  | 77.80 | | 84.78 | 23.1 |
| *R. viscosum* DSM 7307^T^ | 91.11 | 87.70 | 87.68 | 90.36 | 89.77 |  | 76.82 | | 84.27 | 22.1 |
| *B. diazoefficiens* USDA 110 ^T^ | 81.96 | 83.48 | 80.58 | 79.00 | 80.19 |  | 67.77 | | 82.35 | 17.9 |

**Table S8**. Putative genes under positive selection in the *Rhizobium* sp. CACMS001

| Locus | KO annotation | Swissprot annotation |
| --- | --- | --- |
| RCA_00133 | - | Uncharacterized protein YggC |
| RCA_00141 | K00847 | Fructokinase |
| RCA_00187 | K10764 | O-succinylhomoserine sulfhydrylase |
| RCA_00248 | K13582 | Localization factor PodJL |
| RCA_00406 | K00140 | Putative 3-oxopropanoate dehydrogenase |
| RCA_00430 | K01546 | Potassium-transporting ATPase potassium-binding subunit |
| RCA_00543 | - | Uncharacterized protein YciQ |
| RCA_00551 | K06959 | Uncharacterized protein HI_0568 |
| RCA_00615 | K02276 | Cytochrome c oxidase subunit 3 |
| RCA_00686 | K00108 | Oxygen-dependent choline dehydrogenase |
| RCA_00779 | - | - |
| RCA_00794 | - | Na(+)/H(+) antiporter subunit A |
| RCA_00939 | K01649 | (R)-citramalate synthase aeolicus |
| RCA_01051 | K01448 | N-acetylmuramoyl-L-alanine amidase AmiC |
| RCA_01101 | K02907 | 50S ribosomal protein L30 |
| RCA_01246 | K01754 | L-threonine dehydratase biosynthetic IlvA |
| RCA_01283 | K01873 | Valine--tRNA ligase |
| RCA_01794 | K09014 | UPF0051 protein |
| RCA_01840 | K00031 | Isocitrate dehydrogenase [NADP] |
| RCA_01844 | K01872 | Alanine--tRNA ligase |
| RCA_01870 | - | - |
| RCA_02343 | K00548 | Methionine synthase |
| RCA_02397 | K02316 | DNA primase coli |
| RCA_02454 | K00948 | Ribose-phosphate pyrophosphokinase |
| RCA_02489 | - | - |
| RCA_02521 | K13503 | Anthranilate synthase |
| RCA_03020 | K05524 | Ferredoxin-2 |
| RCA_03121 | K06207 | 50S ribosomal subunit assembly factor BipA |
| RCA_03143 | K00239 | Succinate dehydrogenase flavoprotein subunit |
| RCA_03245 | K00928 | Aspartate kinase |
| RCA_03363 | K01805 | Xylose isomerase etli (strain CFN 42 / ATCC 51251) |
| RCA_03448 | - | Uncharacterized protein Rv2567 |
| RCA_03513 | K11751 | 5'-nucleotidase |
| RCA_03636 | K00831 | Phosphoserine aminotransferase |
| RCA_03698 | K03596 | Elongation factor 4 |
| RCA_03703 | - | Probable adenylyltransferase/sulfurtransferase MoeZ |
| RCA_03722 | K08305 | Membrane-bound lytic murein transglycosylase B |
| RCA_03814 | K23535 | Glucose ABC transporter permease protein |
| RCA_04016 | K21062 | 4-hydroxy-tetrahydrodipicolinate synthase |
| RCA_05155 | K00278 | L-aspartate oxidase |
| RCA_05464 | K02026 | Trehalose/maltose transport system permease protein MalG |

**Table S9**. Putative genes under positive selection in the *Rhizobium* sp. CACMS001

**
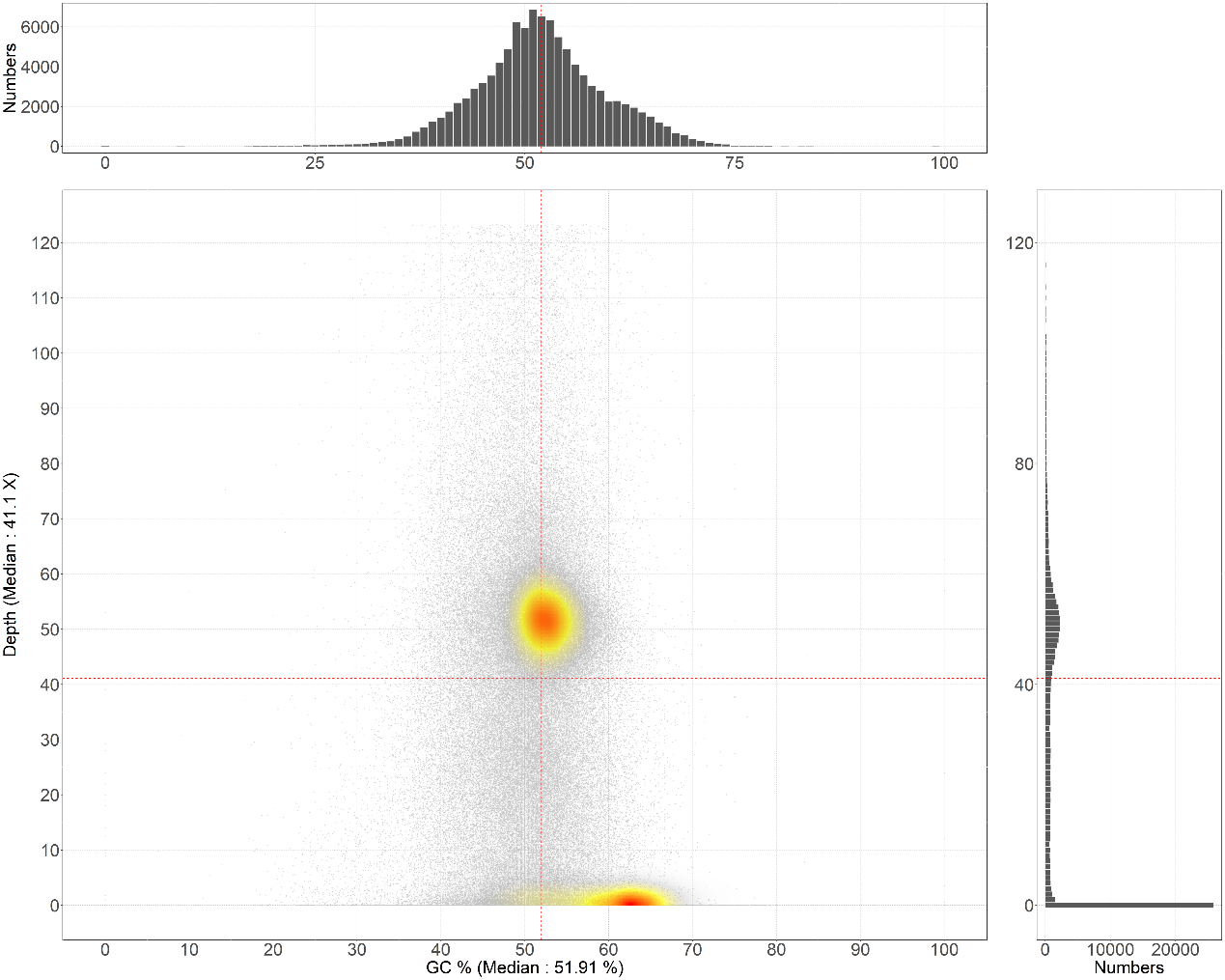
**

**Fig. S1** The estimation of abnormal reads in *Polyporus umbellatus* genome survey sequences. The highlight area at the bottom may be rhizobia. See https://doi.org/10.6084/m9.figshare.19609500 for more details.

**Fig. S2** GO classification statistics of the *Rhizobium* sp. CACMS001

**Fig. S3** KEGG classification statistics of the *Rhizobium* sp. CACMS001

**
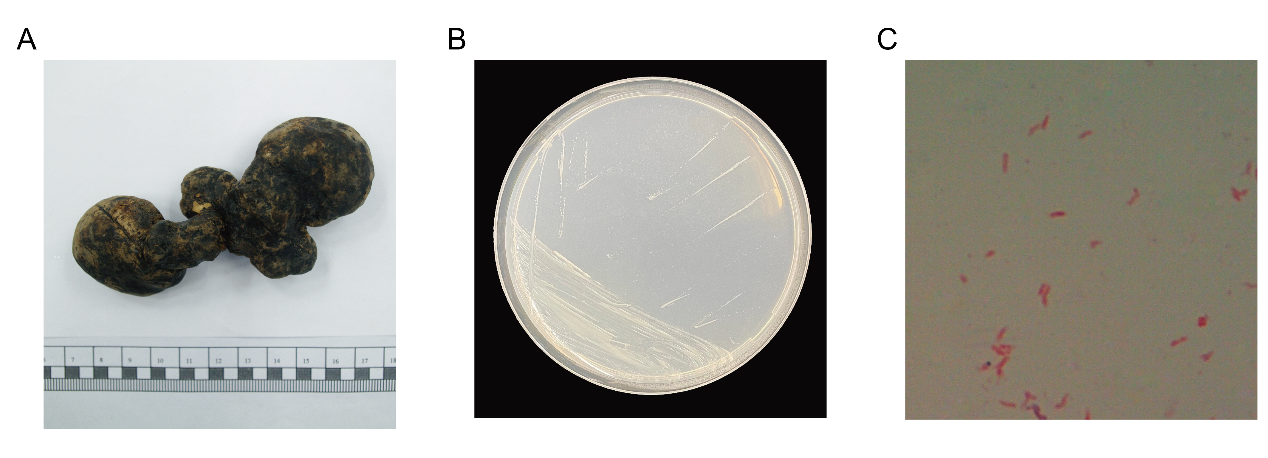
**

**Fig. S4** (A) Host fungi. (B) Colony morphology. (C) Gram stain showing Gram negative


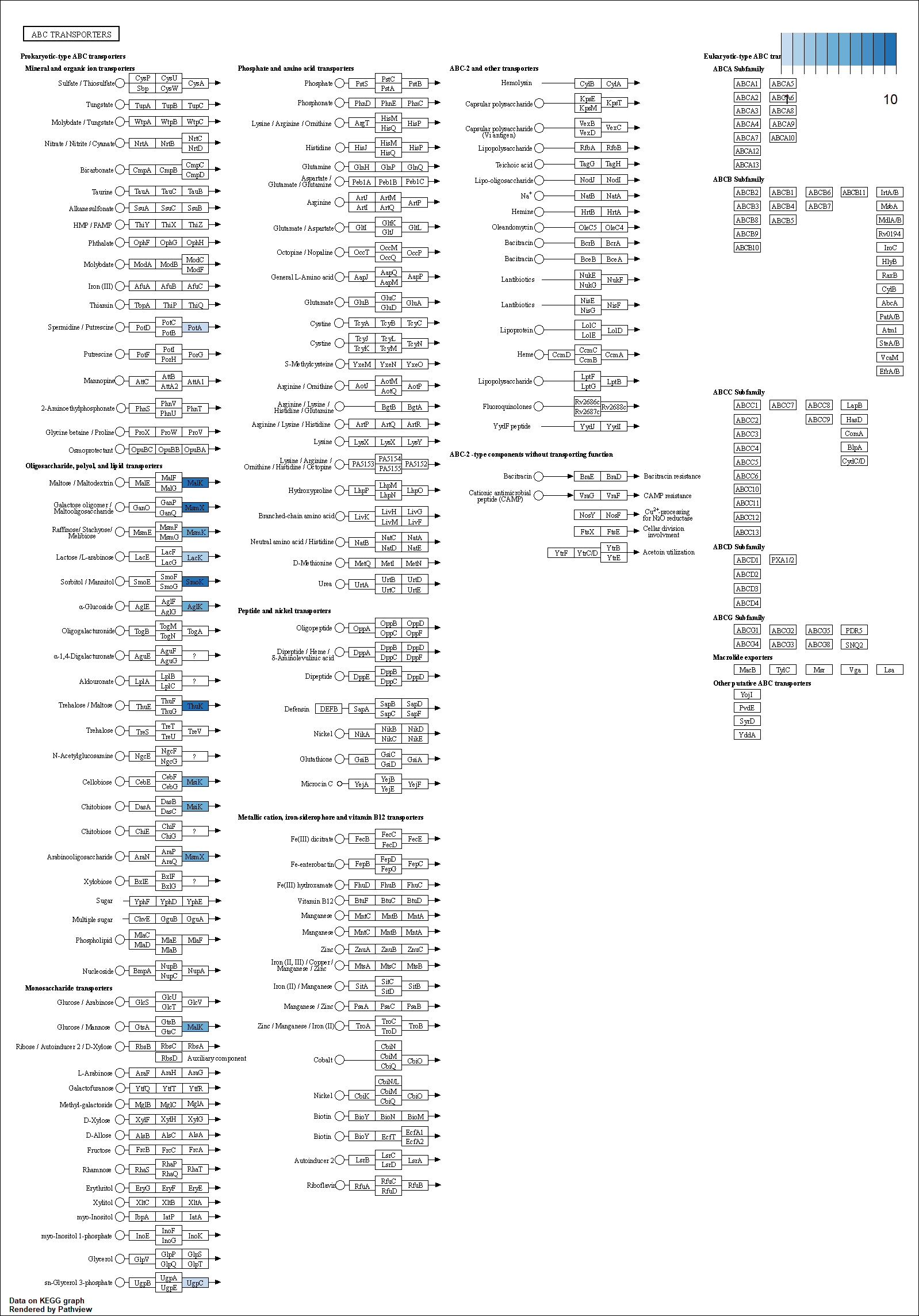


**Fig. S5** ABC transporters in *Rhizobium* sp. CACMS001 species-specific genes


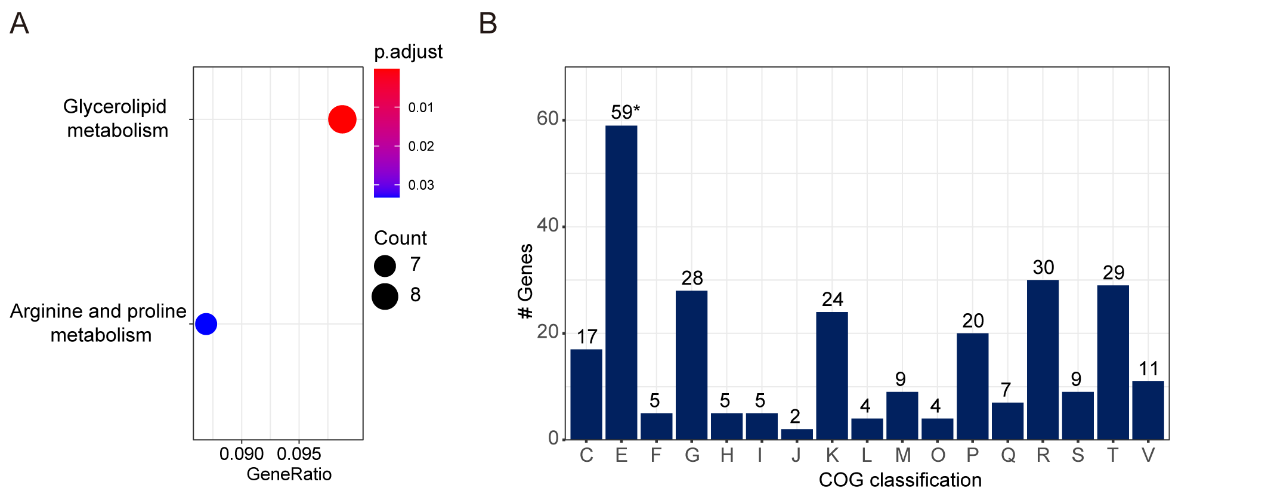


**Fig. S6** KEGG enrichment and COG classification for 95 clusters have more genes in strain CACMS001. **(A)** KEGG enrichment **(B)** COG classification. Asterisk represents significant enrichment with *p* value<0.05.
